# Association between Adverse Effects and Parental Beliefs about Antiepileptic Medicines

**DOI:** 10.3390/medicina54040060

**Published:** 2018-08-28

**Authors:** Violeta Ilić, Dragana Bogićević, Branislava Miljković, Sandra Vezmar-Kovačević

**Affiliations:** 1 Department of Pharmacokinetics and Clinical Pharmacy, Faculty of Pharmacy, University of Belgrade, 11000 Belgrade, Serbia; ilicvioleta@yahoo.com (V.I.); milbran@pharmacy.bg.ac.rs (B.M.); 2 University Children’s Hospital, Faculty of Medicine, University of Belgrade, 11000 Belgrade, Serbia; drbogi.123@gmail.com

**Keywords:** antiepileptic drugs, children, parents, beliefs about medications, adverse events

## Abstract

*Background and Aim:* Adverse effects are common in children treated with antiepileptic medications and may affect parental beliefs about treatment. The aim of the study was to investigate the relationship between adverse effects and parental beliefs about antiepileptic drugs used for the treatment of their children. *Methods:* The study was performed at the University Children’s Hospital, Belgrade, Serbia from 2013–2015. Parents of children treated with valproic acid, carbamazepine or lamotrigine, were eligible. They were asked to fill in the Beliefs about Medications Questionnaire (BMQ) and The Liverpool Adverse Events Profile (LAEP). *Results:* Parents of 127 children (average age 9.88 ± 4.16 years) of whom 111 had epilepsy (67 generalized, 44 focal) and 16 with febrile seizures participated in the study. Nervousness and/or agitation, weight gain, restlessness, headache, difficulty in concentrating, feeling of aggression and upset stomach were most frequent adverse effects, reported in 37% of the population. BMQ-specific necessity scores significantly correlated with parental education; parents with elementary school showed lower scores than those with higher education. The presence of difficulty in concentrating of their child was associated with higher BMQ concern scores (20.73 ± 4.25 vs. 18.99 ± 3.60, *p* = 0.043) as well as necessity scores (18.42 ± 3.31 vs. 16.40 ± 2.73, *p* = 0.017). Higher scores of BMQ-general overuse were reported in the presence of a headache (8.79 ± 2.81 vs. 7.64 ± 2.72, *p* = 0.027). *Conclusions:* The main finding of our study is that parental beliefs about antiepileptic drugs were associated with the presence of adverse effects. Understanding this relationship could allow physicians and pharmacists to structure better educational programs for parents of children treated with antiepileptic drugs. Education should be more focused towards understanding the adverse effects of antiepileptics which could alleviate parental concerns and strengthen their beliefs about the necessity of medication use in their children.

## 1. Introduction

Antiepileptic drugs are commonly prescribed to control epileptic seizures. They are associated with adverse effects in approximately 50% of paediatric patients on monotherapy [1,2]. The most commonly used antiepileptic drugs such as valproic acid, carbamazepine and lamotrigine have been associated with gastrointestinal disturbances, loss of appetite and nausea, weight gain, tremor, rash and fatigue/tiredness [3]. However, the reports of adverse effect prevalence of antiepileptic drugs are inconsistent, probably due to the different methodology used for assessing adverse effects such as spontaneous reporting or specially designed questionnaires for side effects [4]. Whereas some adverse effects of antiepileptics are readily assessed (weight gain/loss, rash), others are difficult to observe and rely on subjective patient reporting [5]. The Liverpool Adverse Events Profile (LAEP) has been developed with the aim to quantify patients’ perceptions of adverse effects of antiepileptic drugs [6,7,8,9]. LAEP is a validated 19-item questionnaire commonly used for quantification of adverse effects of most frequently used antiepileptic drugs [5,10,11,12,13,14].

Medications used in treatment of epilepsy have been related to nonadherence and reduced quality of life, particularly cognitive and neurological impairment [15,16]. Nonadherence has been associated with perceptual barriers such as concerns about adverse effects, doubts about the need for medicines or gaps in knowledge [17,18]. Horne et al. postulated that adherence to treatments is the result of the interplay between patients’ beliefs about the necessity of therapy and their concerns about the potential adverse consequences of treatment [19].

The adherence of young children to medications depends on their parents’ or caregivers’ decisions to administer medicines appropriately. Various barriers to medication adherence for children and their parents have been reported. Parental lack of understanding the disease, worries about the effectiveness and side effects of medications, polypharmacy, the length of treatment duration is some of the reasons for nonadherence [20,21]. Moreover, adverse effects of antiepileptic drugs may be a reason for a parent to discontinue treatment, at least for some time, if the child has well-controlled seizure disorders [16].

The aim of this study was to investigate how the presence of adverse effects of antiepileptic drugs influences parental beliefs about medications.

## 2. Methods

### 2.1. Patient’s Characteristics

The study was performed at the University Children’s Hospital in Belgrade, Serbia during the period 2013–2015. The study was approved by the Ethics Committee of the University Children’s Hospital (Nr 017/6, 26/56 Date 18 March 2013). The research was performed according to the Code of Ethics (Declaration of Helsinki). All participants gave their informed consent before they were included in the study.

Parents of children, aged 0–18 years diagnosed with epilepsy or febrile seizures, who were treated with valproic acid, lamotrigine or carbamazepine for at least six months, were included in the study after signing the informed consent. These drugs were selected since they were most frequently used, as monotherapy, in patients with seizures in our hospital. The main inclusion criteria were: good seizure control of the child with monotherapy, healthy neurological and cognitive development, normal school function (if applicable), normal magnetic resonance imaging scan, normal computed tomography and no other severe co-morbidities (endocrinopathies, liver or kidney diseases, systemic disorders). All patients who fulfilled the inclusion criteria were eligible for the study.

The following data were obtained from the patients: gender, age, seizure type, duration of therapy, drug dose and serum concentration level of the drug measured on the day that the parent completed the questionnaire. Parents were asked about their age, gender and educational attainment.

All participants were parents of ambulatory patients and they had been invited to complete the LAEP and Beliefs about Medicines Questionnaire (BMQ) during a control visit at the hospital.

### 2.2. LAEP

The LAEP is a self-report questionnaire developed to assess the frequency of side effects of antiepileptic drugs. It includes 19 items presented as a checklist of symptoms that may have occurred in the last four weeks. The frequency of side effects is rated on a 4-point Likert scale with 1- never a problem; 2- rarely a problem; 3- sometimes a problem; and 4- always a problem [6,8]. Results can be individually assessed or added up to total scores which range from 19–76 [5,22]. High scores indicate more frequent symptom reporting. The LAEP was translated into the Serbian language. Translations were then evaluated by a group of six native speakers to ensure that the resulting translations are correct, clear and understandable. Back-translation into English was performed by two independent translators native in the target language.

### 2.3. BMQ

In order to examine parental views about their child’s treatment, we used the validated BMQ [19,23]. The BMQ is an 18-item questionnaire, divided into two sections BMQ-Specific and BMQ-General.

The BMQ-Specific was used to measure parental beliefs about the necessity for treatment with antiepileptic drugs as well as concerns or worries associated with their use. Parents were instructed to answer questions about their child’s treatment. The two subscales of BMQ-Specific questionnaire, necessity of medication use and concerns are each assessed by five questions. Necessity is assessed through questions such as “My child’s health at present depends on these medications” and an example of concerns questions is “I sometimes worry about the long-term effects of these medicines on my child.”

The BMQ-General was used to assess more general beliefs about medications [19]. The BMQ-General is divided into General Harm associated with medicines use (five questions, that is, “Medicines do more harm than good”) and General Overuse (three questions, that is, “Doctors place too much trust in medicines”).

All items were scored on a 5-point Likert scale, ranging from strongly disagree (1 point) to strongly agree (5 points). Each BMQ subscale was summed and the higher scores represented stronger beliefs in every subscale. The subscales were also transformed into dichotomous variables where the summed score is split at the midpoint [19]. Scores above the midpoint indicated stronger beliefs in each subscale.

A Necessity-Concerns Differential was calculated as a difference between BMQ-Necessity and BMQ-Concerns scores. This differential can be thought of as an individual cost-benefit analysis where costs are represented by concerns and weighed against benefit (perceived as necessity beliefs) [19].

The BMQ scales were originally developed for adults. However, they have been used to measure parental views about needs and concerns related to their child’s medications [16,24,25,26]. Internal consistency was tested in our sample and Cronbach’s alpha was between 0.65–0.81 in all subscales. In order to assess internal reliability of the BMQ questionnaire we conducted confirmatory Factor analysis through Principal Component Analysis. The Kaiser-Meyer-Olkin Measure of Sampling Adequacy is a statistic that indicates the proportion of variance in variables that might be caused by underlying factors. Since we obtained a high statistic value of 0.753 we are confident that Factor analysis is suitable for analysis. Using Varimax rotation on extracted factors, we obtained factors that fit well with the original model (0.62–0.81) and therefore we continued our analysis in line with the proposed sub-scales (BMQ necessity, BMQ concerns, BMQ overuse, BMQ harm).

### 2.4. Data Management and Statistical Analysis

Statistical analysis was performed using PASW 18 (SPSS Inc., Chicago, IL, USA). Descriptive statistics were carried out to characterize the frequency of the variables. Data were expressed as mean ± standard deviation (SD) values or median and interquartile range (IQR). Mann-Whitney U-test was used to compare BMQ scores. Bivariate Pearson’s correlation was used to assess correlations between BMQ and LAEP scores. Linear regression was used to explore the impact of age (0–18 years), gender, seizure type, medication, duration of treatment and parental gender and education level on LAEP and BMQ scores. The coefficient of determination (R^2^), the regression coefficient (B), its 95% confidence interval (CI) and standard error (SE) were used to describe the linear regression model. Statistical significance was set at *p* < 0.05.

## 3. Results

### 3.1. General Characteristics

In total, parents of 132 children were eligible for the study. Written consent was obtained from 127 (96.1%) parents and they were included in the study. Out of the 127 parents who filled in the questionnaires 106 (83.5%) were mothers and the average age of parents was 36 ± 12 years. The majority of children (111, 87.4%) were diagnosed with epilepsy and 16 (12.6%) had febrile seizures. Sixty-seven patients were diagnosed with generalized onset seizures, (of whom 52 had tonic-clonic seizures, whereas 15 had absence seizures) and 44 patients were diagnosed with focal onset seizures (15 had focal aware seizures, 29 developed focal to bilateral tonic-clonic seizures). The mean daily dose was 589 ± 194 mg of valproic acid, 576 ± 215 mg of carbamazepine and 100 (75–125) mg of lamotrigine. Serum concentration levels of the drugs were in the reference range for 110 patients (86.6%). Three patients on valproic acid had elevated concentrations and 14 patients had levels that were below the reference range (11 on valproic acid, one on carbamazepine and two on lamotrigine). All concentrations out of range were within 20% deviation of the reference range. Patient characteristics and parental educational attainment are presented in Table 1.

### 3.2. Adverse Effects

**Figure 1:**
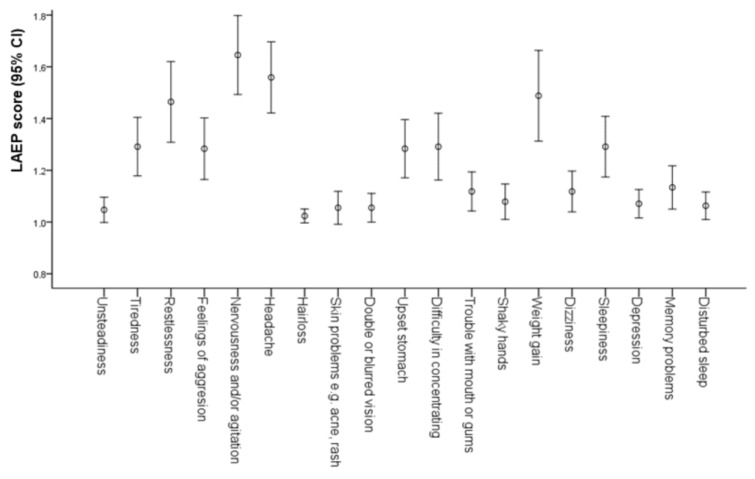
The mean scores of adverse effects as defined by the Liverpool Adverse Event profile (LAEP) with antiepileptic drugs.

The mean LAEP score was 23.36 ± 4.53. The most frequent LAEP-defined specific adverse effects were nervousness and/or agitation (22.9%), weight gain (18.1%), restlessness (17.3%), headache (16.5%), difficulty in concentrating (11.8%), feeling of aggression (11.0%) and upset stomach (10.2%). The mean scores of adverse effects are presented in the Figure 1. There were no statistically significant differences in the overall LAEP score or in specific adverse effects among patients on valproic acid, carbamazepine and lamotrigine. Age, gender, duration of treatment and parental education level and gender had no impact on the LAEP score.

### 3.3. Parental Beliefs about Medications

The mean necessity score in our population was 19.17 ± 3.76. Most parents (91%) had strong beliefs about the need for treatment (BMQ necessity score ≥ 16). The majority of parents (61.4%) also reported strong concerns about their child’s medication and the mean BMQ concerns score was 16.70 ± 2.92. Parents had concerns about potential long-term adverse effects of medication use (65.3% had scores greater than the scale midpoint) and their children becoming dependent upon the medication (53.5%). BMQ concerns score was weakly but significantly correlated with the LAEP score (r_p_ = 0.220; *p* = 0.013).

The mean necessity score was significantly greater than the mean concerns score *p* < 0.001. A mean necessity-concerns differential was 2.47 ± 3.41. The negative necessity-concern differential was reported by 17.3% of parents and same necessity and concerns score was seen in 9.4% of respondents. The BMQ harm score was 12.95 ± 3.33 and the BMQ overuse score was 8 [6,7,8,9,10].

### 3.4. Predictors for BMQ Scores

Parental beliefs about the necessity of their children’s medications were influenced by their education level and the observed difficulty in concentrating of their child. Parents with elementary education had lower BMQ necessity scores compared to parents with university education (18.29 ± 3.37 vs. 20.61 ± 3.20, *p* = 0.04). The presence of difficulty in concentrating increased the necessity scores in comparison to the absence of the adverse effects (20.73 ± 4.25 vs. 18.99 ± 3.60 respectively, *p* = 0.043).

Difficulty in concentrating was also associated with higher concerns score (18.42 ± 3.31 parents who reported difficulty in concentrating versus 16.40 ± 2.73 parents who did not report the adverse effect, *p* = 0.017). Higher scores of medication overuse were reported in the presence of a headache (8.79 ± 2.81 presence of a headache vs. 7.64 ± 2.72 absence of headache, *p* = 0.027). Predictors for BMQ subscale scores are presented in Table 2.

The influence of parental gender on BMQ subscale results was analysed and no statistically significant differences were observed. Nevertheless, linear regression models were performed for fathers and mothers separately. Predictive factors associated with the BMQ subscale results for fathers were not identified. In contrast, the scores of mothers’ BMQ subscales (see Table 3) reflected the overall results presented in Table 2.

## 4. Discussion

The main finding of our study is that parental beliefs about antiepileptic drugs were associated with the presence of adverse effects.

The burden of adverse effects in our cohort was low since the LAEP score did not reach the value of 45 in any patient [22]. Compared to other studies which used the same methodology, we found lower median LAEP scores (22 vs. 30–45 respectively) [5,10,13,14,27,28]. The reason for this discrepancy is probably the fact that in all the aforementioned studies, the participants were adult patients with epilepsy who reported the symptoms themselves. In our study parents were asked to fill in the LAEP questionnaire without previous preparation for the task. Therefore, their responses were based on spontaneous observations and it is possible that they overlooked some symptoms which may have been detected by the patient or a clinician. Moreover, it is possible that the children were not able to recognize or communicate the adverse effects to the parents. However, Harbord reported the presence of significant side effects such as aggression, irritability, hyperactivity, rash, headache, gastrointestinal disturbances and drowsiness in 26% of children with epilepsy [1]. Our results are more consistent with this study since similar adverse effects were reported in 37% of our population. Other factors may have contributed to the lower adverse effect burden such as, our cohort was on monotherapy, they responded to treatment and their serum drug concentration levels were mainly in the reference range. In contrast, studies have shown that lack of 12-month terminal seizure remission, drug resistance and polytherapy contribute to a higher burden of adverse effects [10,27,28]. Most common reported side effects of antiepileptic drugs in the literature were tiredness, memory problems, sleepiness and difficulty in concentrating [5,14,27]. Nervousness and/or agitation, weight gain, restlessness, headache, difficulty in concentrating, feeling of aggression and upset stomach were the most frequent adverse effects observed in our study. A possible reason for the discrepancy of the results may be that tiredness, memory problems and sleepiness may be subjective symptoms that parents may overlook or underestimate, whereas nervousness, agitation, weight gain, may be more easily observed. Kowski et al. associated the use of lamotrigine with difficulties in concentrating, and although a small group of patients in our study was on this medication, our results are in accordance because this was the most common adverse effect reported in this cluster [10].

Most of the parents reported positive beliefs about the necessity of their children’s medication. The necessity score was somewhat higher compared to another study which involved caregivers of children treated with antiepileptic drugs (19.17 ± 3.76 vs. 17.97 ± 4.11, respectively) [16]. BMQ has been used to assess beliefs about medications in parents of asthmatic children and similar results to ours were reported (18.2 ± 3.5) [25]. The mean concern score was also higher compared to the other two studies but the necessity-concerns differential score was similar to the study of asthmatic children, revealing that 25–30% of parents have negative beliefs about their children’s treatment [16,24,25]. This is an important finding because parental concerns about their children’s treatment have been associated with lower levels of adherence [24,29,30]. BMQ harm and overuse score were lower compared to the necessity and concern scores but this was expected since patients were on monotherapy.

Age and gender of the child, duration of treatment, seizure type and medication were not associated with BMQ subscale scores. Since we found no other study that associates adverse effects of antiepileptic drugs with parental beliefs about medicines a direct comparison is not possible. Moreover, we also found no other studies addressing parental beliefs about medicines in which the influence of the investigated parameters was reported. However, we cannot exclude the possibility that the number of patients in our study was not sufficient to detect the influence of the investigated parameters.

In contrast, a lower level of parental education was associated with a stronger doubt of the necessity of their children taking medications to treat seizures. Such results are in accordance with previous studies which have demonstrated a similar impact of education level on beliefs about the necessity of medicines use scores [31]. Moreover, parental higher education level was reported to be an independent predictor of higher epilepsy knowledge [32]. Also, inadequate health literacy of parents was associated with lower level of adherence in epilepsy patients [30,33].

Difficulty in concentrating was predictive of higher necessity as well as higher concerns scores. Higher parental BMQ necessity or concerns scores have been associated with asthma symptoms severity in their children [25,26]. However, the unusual finding in our study was that parents seemed to identify difficulty in concentrating as an adverse effect of their child’s medication but this strengthened their belief about the necessity of treatment. A possible explanation could be that parents may have perceived difficulty in concentrating not only as a possible adverse effect but also as a symptom of illness. Having doubts about the nature of the symptom could have strengthened parent’s beliefs about the necessity of treatment concomitantly increasing their concerns. This may be corroborated by the fact that a headache, a common adverse effect of many medications, was associated with beliefs about medication overuse. It may be possible that parents were not concerned about headaches because they recognized them as usual medication side effects and did not perceive them as serious. In contrast, difficulty in concentrating could have raised concerns about their child’s cognitive development which could have further affected their beliefs about the necessity of treatment as well as concerns. Further investigation is needed to elucidate this association.

Although parental gender was not associated with differences in the LAEP score or BMQ subscale results, we analysed mothers’ and fathers’ answers separately. We did not identify predictive factors for fathers’ answers but, female parents’ results were in accordance with the overall model. Our results are in contrast with a study in which male parents expressed more negative beliefs, more abuse beliefs and more beliefs about damage of the medicines to their children than mothers [34]. However, in our study fathers constituted a small portion of the population (16.5%) and we can assume that the number of male parents was insufficient to capture the difference.

There are potential limitations to this study. First, it was a cross-sectional study implying that no conclusions could be drawn with regard to a causal relationship between adverse effects of antiepileptic drugs and parental beliefs and concerns about their child’s medication. Second, only patients on monotherapy with controlled seizures were included in the study which limits generalizability to the population of paediatric patients with seizures. Third, parents were not introduced to the content of LAEP in advance, which could have influenced negatively the number of adverse effects reported in this study. Fourth, we did not investigate the potential impact of parental socioeconomic status on BMQ scores.

## 5. Conclusions

Despite its limitations, this study was the first to quantify adverse effects of antiepileptic drugs linked to parental perceptions about their child’s treatment. The findings indicate that parents observed nervousness and/or agitation, weight gain, restlessness, headache, difficulty in concentrating, feeling of aggression and upset stomach as most frequent adverse effects following monotherapy with valproic acid, lamotrigine and carbamazepine. Parents perceived their child’s medication as necessary but also revealed a high concern score associated with the medication use. Moreover, the presence of difficulty in concentrating raised concerns and strengthened parental beliefs about the necessity of medication use. In contrast, the presence of headaches was associated with parental beliefs about medication overuse. Higher education level of parents affected the beliefs about the necessity of medication use positively.

Further investigation of the relationship between parental beliefs and concerns about medications and the presence of side effects in a randomised controlled trial is necessary. Understanding this relationship could allow physicians and pharmacists to structure better educational programs for parents of children on antiepileptic drugs. Education should be more focused towards understanding the adverse effects of antiepileptic medications which could alleviate parental concerns and strengthen their beliefs about the necessity of medication use in their children.

## Figures and Tables

**Table 1:** Patient characteristics and parental education level.

Patient Characteristics	Total (*N* = 127)
Age, mean ± SD, years	9.88 ± 4.16
Gender, male *n* (%)	64 (50.4)
Treatment *n* (%)	
Valproic acid	102 (80.3)
Carbamazepine	13 (10.2)
Lamotrigine	12 (9.5)
Dose *n* (%)	
Valproic acid	
20 mg/kg	52 (51.0)
25 mg/kg	11 (10.8)
30 mg/kg	3 (2.9)
≥45 kg	36 (35.3)
Carbamazepine	
10 mg/kg	3 (23.1)
20 mg/kg	2 (15.4)
≥45 kg	8 (61.5)
Lamotrigine	
3 mg/kg	9 (75.0)
4 mg/kg	1 (8.3)
≥45 kg	2 (16.7)
Treatment duration, median (IQR), years	3 (2-5)
Parental age, mean ± SD, years	36 ± 12
Gender, female *n* (%)	106 (83.5)
Parental education level *n* (%)	
Primary school	28 (22.0)
Secondary school	83 (65.4)
University	16 (12.6)

**Table 2:** Predictors for Beliefs about Medications Questionnaire (BMQ) subscale scores.

Dependent Value	Predictor	R^2^	B (95% CI)	SE	*p*-Value
BMQ necessity	Parental education level	0.273	1.13 (0.03–2.24)	0.56	0.045
	Difficulty in concentrating	0.273	1.08 (0.21–1.96)	0.44	0.016
BMQ concerns	Difficulty in concentrating	0.262	1.04 (0.36–1.72)	0.34	0.003
BMQ overuse	Headache	0.193	0.70 (0.07–1.32)	0.32	0.029
BMQ-harm	not identified				

The coefficient of determination (R^2^), the regression coefficient (B), standard error (SE).

**Table 3:** Predictors for BMQ subscale results when mothers answered the questionnaire.

Dependent Value	Predictor	R^2^	B (95% CI)	SE	*p*-Value
BMQ necessity	Parental education level	0.300	1.41 (0.20–2.63)	0.61	0.023
	Difficulty in concentrating	0.300	1.03 (0.07–1.99)	0.49	0.036
BMQ concerns	Difficulty in concentrating	0.231	0.86 (0.16–1.56)	0.35	0.017
BMQ overuse	Headache	0.219	0.80 (0.11–1.51)	0.35	0.024
BMQ- harm	not identified				
